# Osimertinib as treatment for EGFR exon 20 insertion-positive lung adenocarcinoma

**DOI:** 10.17179/excli2019-1786

**Published:** 2019-10-07

**Authors:** Chihiro Murano, Akira Igarashi, Keiko Yamauchi, Sumito Inoue, Masafumi Watanabe

**Affiliations:** 1 Postgraduate Clinical Training Center, Yamagata University Hospital, Yamagata, Japan; 2 Department of Cardiology, Pulmonology, and Nephrology, Yamagata University Faculty of Medicine, Yamagata, Japan

**Keywords:** lung cancer, adenocarcinoma, EGFR exon 20 insertion gene mutation, osimertinib

## Abstract

A 20-year-old woman was diagnosed with stage 4 lung adenocarcinoma with an epidermal growth factor receptor (EGFR) exon 20 insertion gene mutation. Although the patient underwent chemotherapy, her lesions progressed. Liquid biopsy for EGFR T790M mutation showed negative results. After administering osimertinib, reduction of the lesions at the primary site was observed, and the patient's respiratory condition improved. Previous reports showing successful treatment of EGFR exon 20 insertion-positive lung adenocarcinoma with the standard osimertinib dose of 80 mg are limited. The present case demonstrated that osimertinib could be a possible treatment option for EGFR exon 20 insertion-positive lung adenocarcinoma.

## Introduction

The frequency of exon 20 insertions in the epidermal growth factor receptor (EGFR) gene has been reported to account for 2.0-5.8 % of the total EGFR mutations. According to previous reports, non-small cell lung cancers with EGFR exon 20 insertions are resistant to EGFR-tyrosine kinase inhibitor (TKI) treatment. Only a small number of cases have been reported in which osimertinib was administered to patients with lung cancer who were positive for EGFR exon 20 insertions (Jänne et al., 2015[3], Piotrowska et al., 2018[6], Riess et al., 2017[7], Veggel et al., 2018[8]). There is currently limited research on the effect of EGFR-TKI on exon 20 insertion positive non-small cell lung cancer. Osimertinib may be an effective treatment for non-small cell lung cancer with exon 20 insertions.

## Case report

A woman in her 20s with no previous history of smoking was diagnosed with stage 4 lung adenocarcinoma (cT2aN3M1b) with associated liver metastasis, multiple bone metastasis, and multiple brain metastasis. Her staging was based on the classification of lung cancer as per The Japan Lung Cancer Society, 7^th^ edition. Gene analysis revealed positive results for an EGFR exon 20 insertion mutation and negative results for an echinoderm microtubule-associated protein-like 4 anaplastic lymphoma kinase (EML4-ALK) gene fusion. PD-L1 expression in the lung biopsy tissue was 80-90 %.

**Figure 1:**
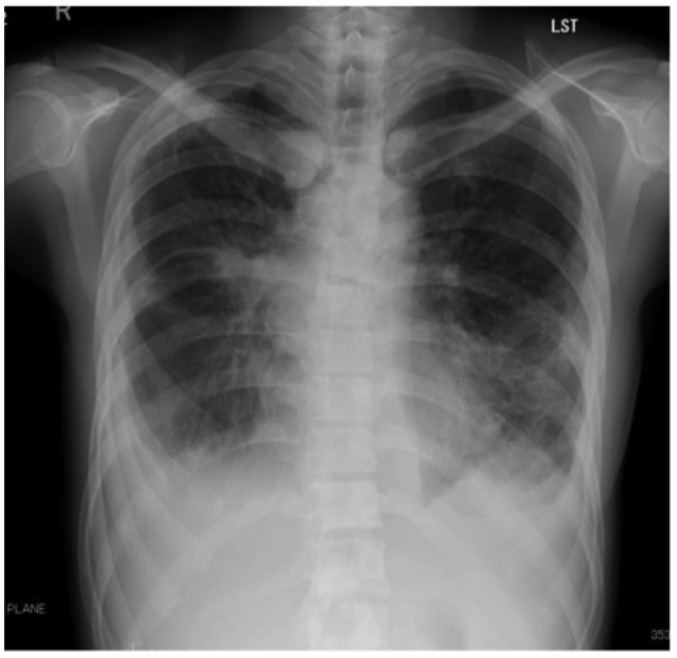
Chest X-ray scan revealed a tumor shadow in the right hilar area, an invasive shadow in the left middle and lower lung fields, and pleural effusion on both sides.

She received cisplatin and pemetrexed sodium hydrate (PEM) chemotherapy. Following single-agent PEM chemotherapy administration, we observed tumor progression and an increasing number of cysts in the lung. Thoracoscopic lung biopsy revealed multiple metastatic lesions in the lung field and cystic lesions associated with the check valve mechanism containing cancer cells, but no evidence of interstitial pneumonia or vasculitis was noted. There were no findings in the laboratory data suggesting any collagenous diseases. In September X+1 year, she was administered 30 mg of afatinib orally, but administration was discontinued after 30 days due to hepatic dysfunction. After her hepatic function improved, she received 20 mg of afatinib; however, a liver function disorder was observed 5 days after the afatinib administration. Due to hepatic dysfunction and disease progression, afatinib was discontinued. In November X+1 year, she received docetaxel hydrate (DTX) without ramucirumab, because of the high risk of bleeding from the bronchus. Even though the treatment effects of DTX were obtained, her treatment was changed to pembrolizumab because it was commercially available. However, tumor progression was observed even though three courses of pembrolizumab treatment were administered. DTX was administered again from June to August X year, and tumor growth was still observed despite the total eight courses of treatment. In September, the patient received one course of amrubicin, but it was ineffective. Because of an eating disorder, malaise, and breathing difficulty, the patient was readmitted to the hospital in October (2 years from the diagnosis). Blood biochemical examination findings at this time revealed the following: leukocyte increased to 16400/μl (neutrophils, 91.0 %; lymphocytes, 4.1 %; monocytes, 4.4 %; eosinophils, 0.10 %; and basophils, 0.40 %), hemoglobin level reduced to 10.5 g/dl, C-reactive protein level increased to 16.93 mg/dl, and CYFRA level increased to 17.94 ng/dl. The results of the EGFR mutation plasma test were negative for the T790M mutation and positive for the exon 20 insertion. Histological examination to assess the EGFR mutation could not be performed due to her poor general condition. A chest X-ray (Figure 1(Fig. 1)) revealed a tumor shadow in the right hilar area, an invasive shadow in the left middle and lower lung fields, and pleural effusion on both sides. 

**Figure 2:**
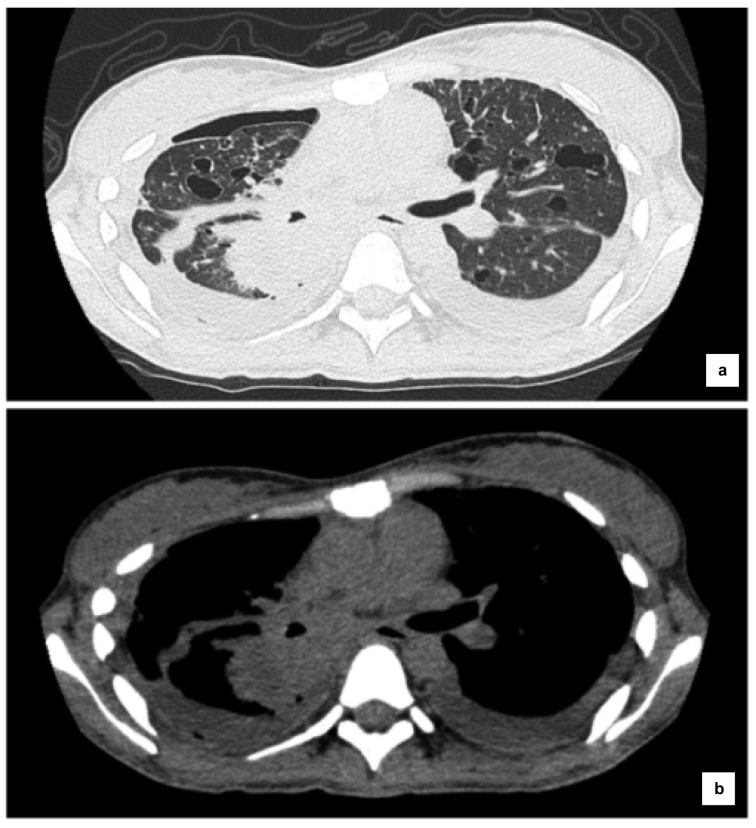
Computed tomography (CT) scan before administration of osimertinib. Primary lesions in the right middle and lower lobes, multiple cystic lesions in the lung field, and mild pneumothorax were observed in the right lung (a). Pleural effusion on both sides was observed (b).

Computed tomography (CT) (Figure 2(Fig. 2)) revealed tumors in the right middle and lower lobes, multiple cystic lesions in the lung field, mild pneumothorax of the right lung (Figure 2a(Fig. 2)), and pleural effusion on both sides (Figure 2b(Fig. 2)).

**Figure 3:**
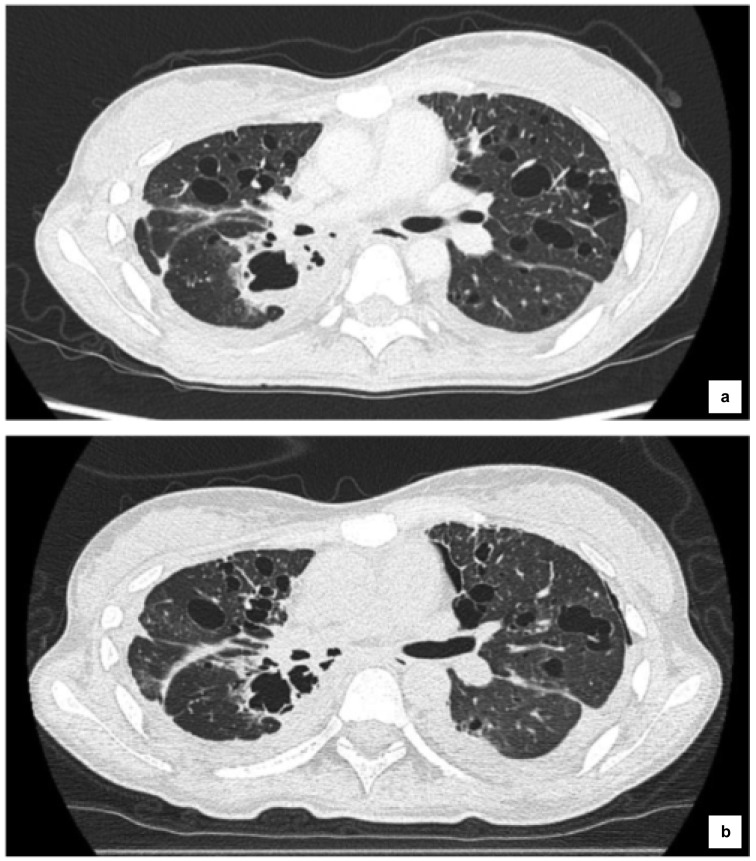
CT images after administration of osimertinib. Cavitation and decrease in size of the primary lesion were observed after 43 (a) and 81 days (b).

After hospitalization, deterioration of her respiratory condition and performance status (PS) was observed due to disease progression (PS=3). A standard dose (80 mg) of osimertinib was administered after explaining the effects and side effects of the treatment to the patient and her family. Respiratory state improved, and PS improved from 3 to 1, approximately 1 week from the introduction of osimertinib; cavitation and decrease in size of the primary lesion were observed via CT after 43 (Figure 3a(Fig. 3)) and 81 days (Figure 3b(Fig. 3)), respectively. The maximum effect was judged as a partial response (PR). CYFRA level decreased to 6.02 ng/dl, and CRP level decreased to 0.43 mg/dl at 16 days from the administration of osimertinib. Approximately 4 months after initiating osimertinib treatment, her respiratory condition deteriorated due to hemoptysis, and the patient died approximately 2 years and 5 months after the initial diagnosis of lung cancer.

## Discussion

To the best of our knowledge, there is only a small number of case reports that demonstrate successful treatment of EGFR exon 20 insertion-positive lung adenocarcinoma with a standard osimertinib dose of 80 mg (Jänne et al., 2015[3], Piotrowska et al., 2018[6], Riess et al., 2017[7], Veggel et al., 2018[8]). Exon 19 deletion mutations and exon 21 L858R mutations constitute approximately 90 % of EGFR mutations (Beau-Faller et al., 2014[1]). Other gene mutations, generally known as uncommon mutations such as E709X, G719X, S768I, P848L, L861Q, and exon 20 insertions, are located in exons 18-21. Exon 20 insertions have been reported in 2.0-5.8 % of EGFR mutation cases; such cases are considered to be resistant to EGFR-TKI (Kobayashi and Mitsudomi, 2016[4]; Yasuda et al., 2012[9]). In previous reports, one patient with exon 20 insertions received 40 mg osimertinib in the AURA test (Phase I), but disease progression was observed (Jänne et al., 2015[3]). Two other reports showed partial responses with 160 mg osimertinib used for treating patients with exon 20 positive insertions (Piotrowska et al., 2018[6]; Riess et al., 2017[7]). However, interstitial pneumonia was observed, and therefore, administration was discontinued (Riess et al., 2017[7]). In 2018, Veggel et al. reported the standard dose effect of osimertinib for patients with EGFR exon 20 insertion-positive non-small-cell lung cancer (Veggel et al., 2018[8]). They observed only one patient with partial response. The objective response rate was reported to be 6 %.

Several genetic variants have been reported in EGFR exon 20 insertion mutations (Kobayashi and Mitsudomi, 2016[4]; Oxnard et al., 2013[5]; Yasuda et al., 2012[9], 2013[10]). Genetic variants sensitive to first-generation EGFR-TKIs have also been observed (Kobayashi and Mitsudomi, 2016[4]). Although the gene mutation type of exon 20 insertions in this case is unknown, we believe that it is not a type of mutation that demonstrates the effects of first-generation EGFR-TKIs. Hirano et al. examined the effect of EGFR-TKI on culture cell lines transfected with exon 20 insertion mutations. The IC_50_ value of osimertinib was lower than that of other EGFR-TKIs in any cell lines. For exon 20 insertions, it is suggested that osimertinib may be more effective than other EGFR-TKIs (Hirano et al., 2015[2]). In this case, afatinib was administered only for 1 month due to liver dysfunction; owing to a short period of afatinib administration, the effect was not observed. The T790M mutation was not detected via the plasma test before the administration of osimertinib. Although tissue specimens could not be studied immediately before administering osimertinib, we believe that a possible T790M mutation being induced by afatinib is very low.

Although exon 20 insertions are known to be resistant to EGFR-TKIs, osimertinib may be effective. Clinical trials are currently underway to examine the effects of several EGFR-TKIs on non-small cell lung cancer positivity for exon 20 insertion mutations (UMIN000031929, ClinicalTrials.gov Identifier: NCT03066206, ClinicalTrials.gov Identifier: NCT03414814). We hope that these results will establish evidence that osimertinib and other EGFR-TKIs are effective for non-small cell lung cancer positivity for exon 20 insertion mutations.

## Acknowledgement

We would like to thank Editage (www.editage.jp) for English language editing.

## Conflict of interest

We have no financial relationship with the organization that sponsored the research.
